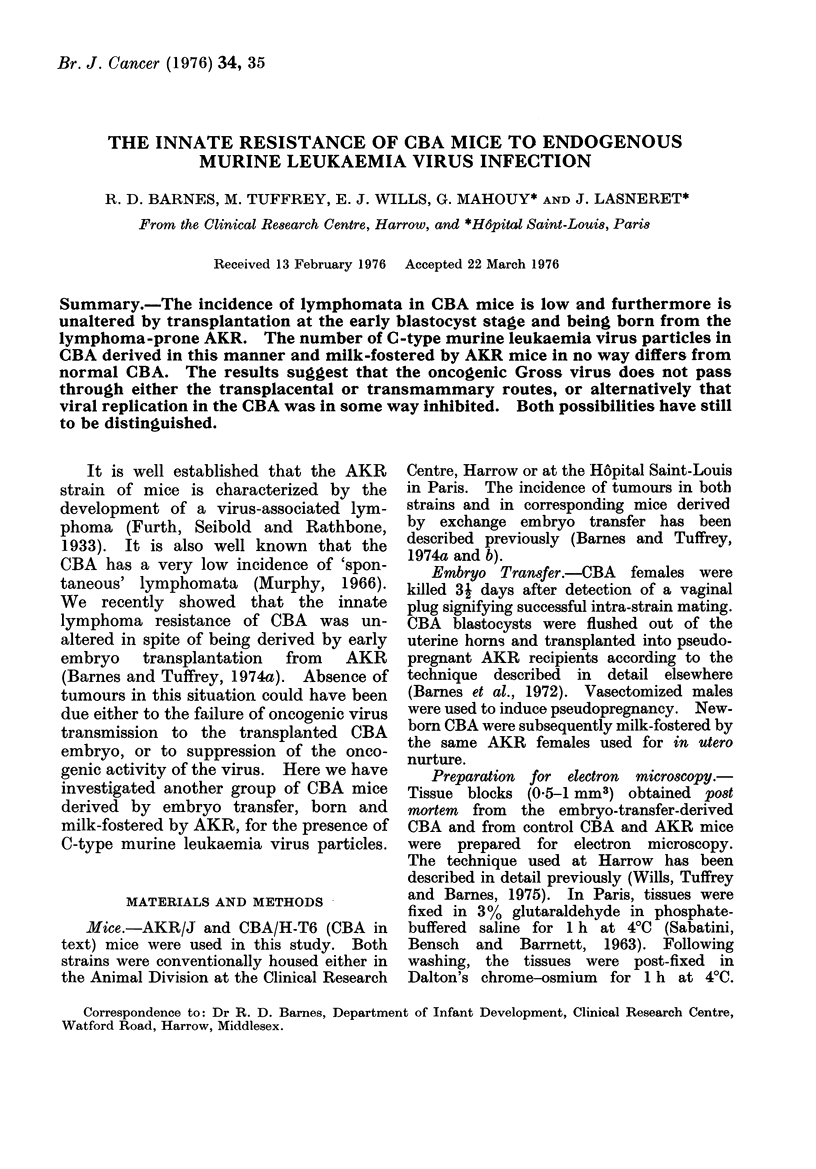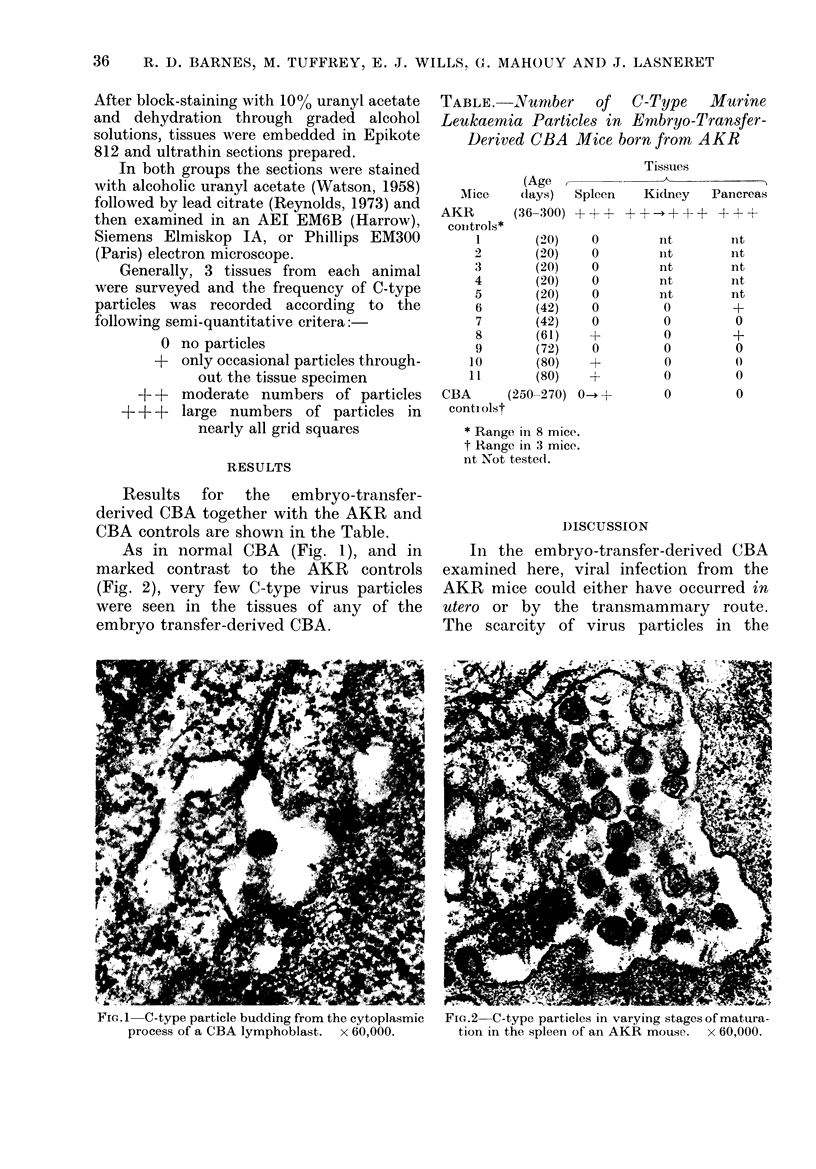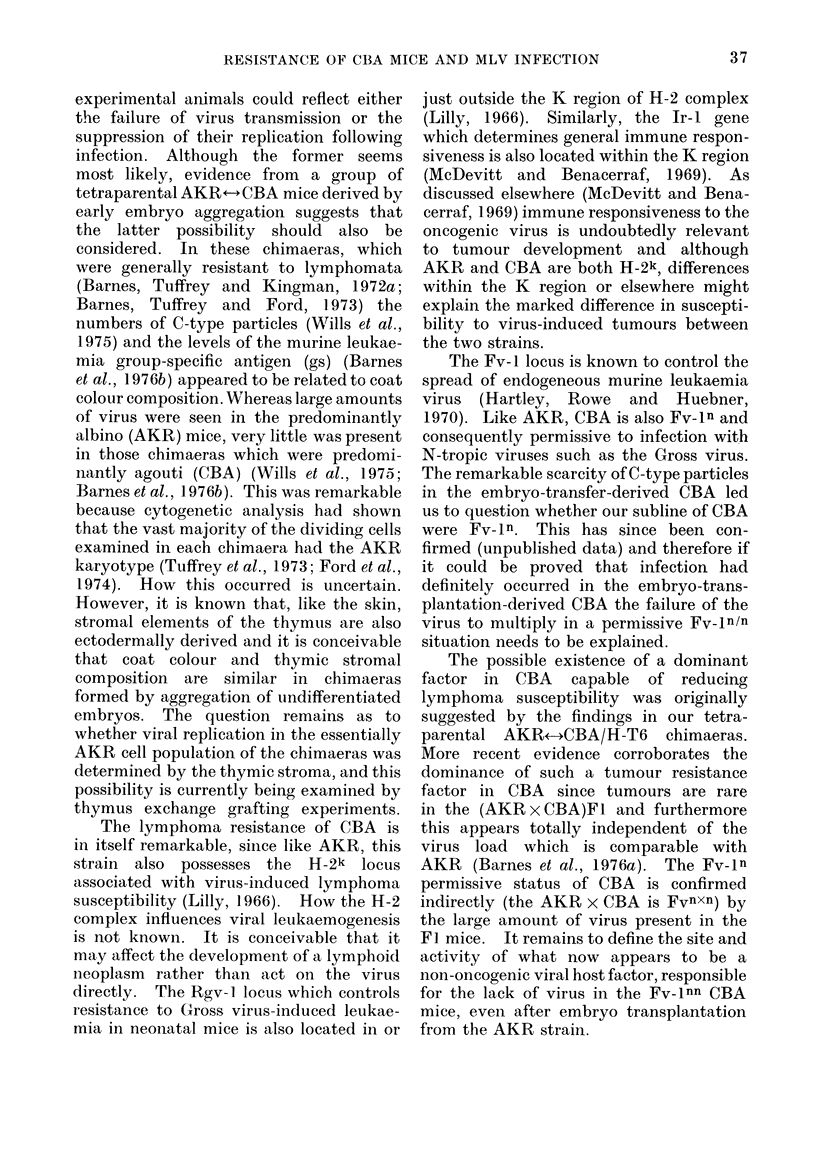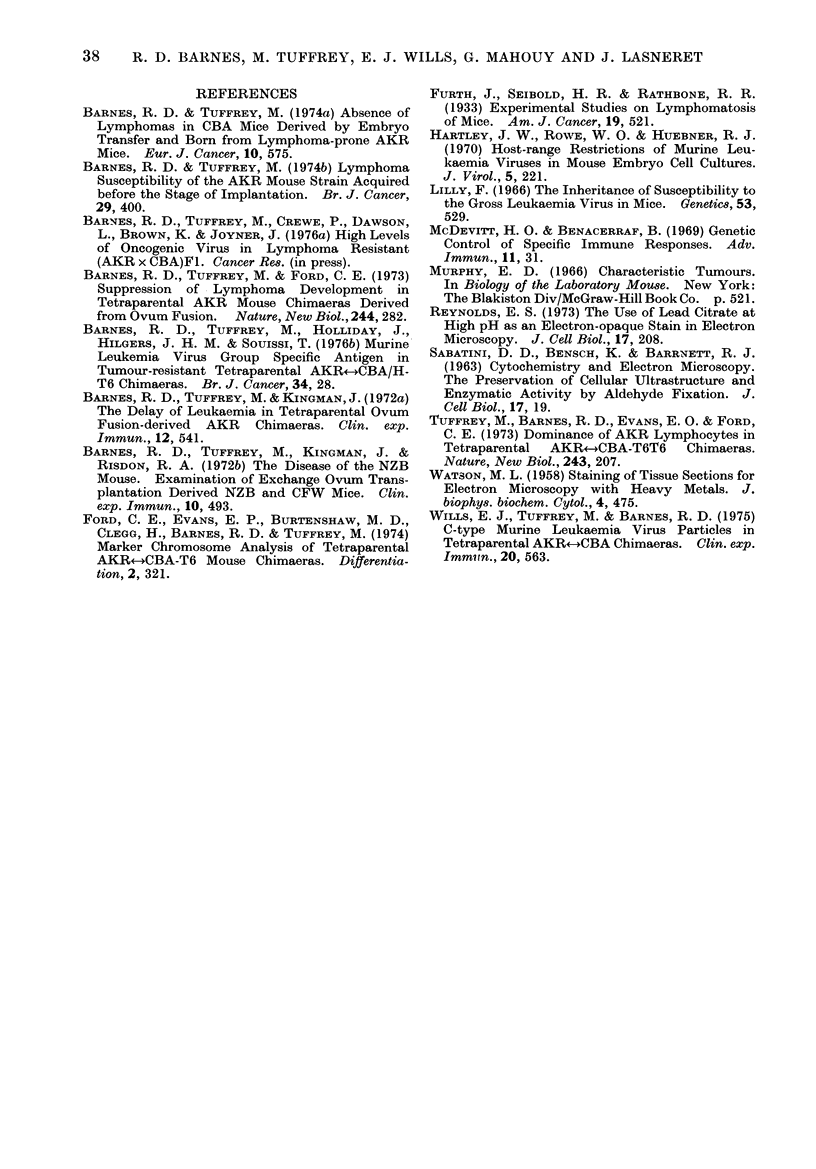# The innate resistance of CBA mice to endogenous murine leukaemia virus infection.

**DOI:** 10.1038/bjc.1976.118

**Published:** 1976-07

**Authors:** R. D. Barnes, M. Tuffrey, E. J. Willis, G. Mahouy, J. Lasneret

## Abstract

**Images:**


*Br. J. Cancer* (1976) **34,** 35

### THE INNATE RESISTANCE OF CBA MICE TO ENDOGENOUS MURINE LEUKAEMIA VIRUS INFECTION

R. D. BARNES, M. TUFFREY, E. J. WILLS, G. MAHOUY* AND J. LASNERET*

*From the Clinical Research Centre, Harrow, and *Hôpital Saint-Louis, Paris*

Received 13 February 1976 Accepted 22 March 1976

**Summary.—The incidence of lymphomata in CBA mice is low and furthermore is unaltered by transplantation at the early blastocyst stage and being born from the lymphoma-prone AKR. The number of C-type murine leukaemia virus particles in CBA derived in this manner and milk-fostered by AKR mice in no way differs from normal CBA. The results suggest that the oncogenic Gross virus does not pass through either the transplacental or transmammary routes, or alternatively that viral replication in the CBA was in some way inhibited. Both possibilities have still to be distinguished.**

It is well established that the AKR strain of mice is characterized by the development of a virus-associated lymphoma (Furth, Seibold and Rathbone, 1933). It is also well known that the CBA has a very low incidence of 'spontaneous' lymphomata (Murphy, 1966). We recently showed that the innate lymphoma resistance of CBA was unaltered in spite of being derived by early embryo transplantation from AKR (Barnes and Tuffrey, 1974*a*). Absence of tumours in this situation could have been due either to the failure of oncogenic virus transmission to the transplanted CBA embryo, or to suppression of the oncogenic activity of the virus. Here we have investigated another group of CBA mice derived by embryo transfer, born and milk-fostered by AKR, for the presence of C-type murine leukaemia virus particles.

### MATERIALS AND METHODS

*Mice.*—AKR/J and CBA/H-T6 (CBA in text) mice were used in this study. Both strains were conventionally housed either in the Animal Division at the Clinical Research Centre, Harrow or at the Hôpital Saint-Louis in Paris. The incidence of tumours in both strains and in corresponding mice derived by exchange embryo transfer has been described previously (Barnes and Tuffrey, 1974*a* and *b*).

*Embryo Transfer.*—CBA females were killed 3½ days after detection of a vaginal plug signifying successful intra-strain mating. CBA blastocysts were flushed out of the uterine horns and transplanted into pseudopregnant AKR recipients according to the technique described in detail elsewhere (Barnes *et al.*, 1972). Vasectomized males were used to induce pseudopregnancy. Newborn CBA were subsequently milk-fostered by the same AKR females used for *in utero* nurture.

*Preparation for electron microscopy.*—Tissue blocks (0·5–1 $mm^3$) obtained *post mortem* from the embryo-transfer-derived CBA and from control CBA and AKR mice were prepared for electron microscopy. The technique used at Harrow has been described in detail previously (Wills, Tuffrey and Barnes, 1975). In Paris, tissues were fixed in 3% glutaraldehyde in phosphate-buffered saline for 1 h at 4°C (Sabatini, Bensch and Barrnett, 1963). Following washing, the tissues were post-fixed in Dalton's chrome–osmium for 1 h at 4°C.

Correspondence to: Dr R. D. Barnes, Department of Infant Development, Clinical Research Centre, Watford Road, Harrow, Middlesex.

After block-staining with 10% uranyl acetate and dehydration through graded alcohol solutions, tissues were embedded in Epikote 812 and ultrathin sections prepared.

In both groups the sections were stained with alcoholic uranyl acetate (Watson, 1958) followed by lead citrate (Reynolds, 1973) and then examined in an AEI EM6B (Harrow), Siemens Elmiskop IA, or Phillips EM300 (Paris) electron microscope.

Generally, 3 tissues from each animal were surveyed and the frequency of C-type particles was recorded according to the following semi-quantitative critera:—

0 no particles
+ only occasional particles throughout the tissue specimen
++ moderate numbers of particles
+++ large numbers of particles in nearly all grid squares

### RESULTS

Results for the embryo-transfer-derived CBA together with the AKR and CBA controls are shown in the Table.

As in normal CBA (Fig. 1), and in marked contrast to the AKR controls (Fig. 2), very few C-type virus particles were seen in the tissues of any of the embryo transfer-derived CBA.

TABLE.—*Number of C-Type Murine Leukaemia Particles in Embryo-Transfer-Derived CBA Mice born from AKR*

| Mice | (Age days) | Tissues: Spleen | Kidney | Pancreas |
|---|---|---|---|---|
| AKR controls* | (36–300) | +++ | ++→+++ | +++ |
| 1 | (20) | 0 | nt | nt |
| 2 | (20) | 0 | nt | nt |
| 3 | (20) | 0 | nt | nt |
| 4 | (20) | 0 | nt | nt |
| 5 | (20) | 0 | nt | nt |
| 6 | (42) | 0 | 0 | + |
| 7 | (42) | 0 | 0 | 0 |
| 8 | (61) | + | 0 | + |
| 9 | (72) | 0 | 0 | 0 |
| 10 | (80) | + | 0 | 0 |
| 11 | (80) | + | 0 | 0 |
| CBA controls† | (250–270) | 0→+ | 0 | 0 |

* Range in 8 mice.

† Range in 3 mice.

nt Not tested.

### DISCUSSION

In the embryo-transfer-derived CBA examined here, viral infection from the AKR mice could either have occurred *in utero* or by the transmammary route. The scarcity of virus particles in the

FIG. 1—C-type particle budding from the cytoplasmic process of a CBA lymphoblast. ×60,000.

FIG. 2—C-type particles in varying stages of maturation in the spleen of an AKR mouse. ×60,000.

experimental animals could reflect either the failure of virus transmission or the suppression of their replication following infection. Although the former seems most likely, evidence from a group of tetraparental AKR↔CBA mice derived by early embryo aggregation suggests that the latter possibility should also be considered. In these chimaeras, which were generally resistant to lymphomata (Barnes, Tuffrey and Kingman, 1972*a*; Barnes, Tuffrey and Ford, 1973) the numbers of C-type particles (Wills *et al.*, 1975) and the levels of the murine leukaemia group-specific antigen (gs) (Barnes *et al.*, 1976*b*) appeared to be related to coat colour composition. Whereas large amounts of virus were seen in the predominantly albino (AKR) mice, very little was present in those chimaeras which were predominantly agouti (CBA) (Wills *et al.*, 1975; Barnes *et al.*, 1976*b*). This was remarkable because cytogenetic analysis had shown that the vast majority of the dividing cells examined in each chimaera had the AKR karyotype (Tuffrey *et al.*, 1973; Ford *et al.*, 1974). How this occurred is uncertain. However, it is known that, like the skin, stromal elements of the thymus are also ectodermally derived and it is conceivable that coat colour and thymic stromal composition are similar in chimaeras formed by aggregation of undifferentiated embryos. The question remains as to whether viral replication in the essentially AKR cell population of the chimaeras was determined by the thymic stroma, and this possibility is currently being examined by thymus exchange grafting experiments.

The lymphoma resistance of CBA is in itself remarkable, since like AKR, this strain also possesses the $H\text{-}2^k$ locus associated with virus-induced lymphoma susceptibility (Lilly, 1966). How the H-2 complex influences viral leukaemogenesis is not known. It is conceivable that it may affect the development of a lymphoid neoplasm rather than act on the virus directly. The Rgv-1 locus which controls resistance to Gross virus-induced leukaemia in neonatal mice is also located in or just outside the K region of H-2 complex (Lilly, 1966). Similarly, the Ir-1 gene which determines general immune responsiveness is also located within the K region (McDevitt and Benacerraf, 1969). As discussed elsewhere (McDevitt and Benacerraf, 1969) immune responsiveness to the oncogenic virus is undoubtedly relevant to tumour development and although AKR and CBA are both $H\text{-}2^k$, differences within the K region or elsewhere might explain the marked difference in susceptibility to virus-induced tumours between the two strains.

The Fv-1 locus is known to control the spread of endogeneous murine leukaemia virus (Hartley, Rowe and Huebner, 1970). Like AKR, CBA is also $Fv\text{-}1^n$ and consequently permissive to infection with N-tropic viruses such as the Gross virus. The remarkable scarcity of C-type particles in the embryo-transfer-derived CBA led us to question whether our subline of CBA were $Fv\text{-}1^n$. This has since been confirmed (unpublished data) and therefore if it could be proved that infection had definitely occurred in the embryo-transplantation-derived CBA the failure of the virus to multiply in a permissive $Fv\text{-}1^{n/n}$ situation needs to be explained.

The possible existence of a dominant factor in CBA capable of reducing lymphoma susceptibility was originally suggested by the findings in our tetraparental AKR↔CBA/H-T6 chimaeras. More recent evidence corroborates the dominance of such a tumour resistance factor in CBA since tumours are rare in the (AKR×CBA)F1 and furthermore this appears totally independent of the virus load which is comparable with AKR (Barnes *et al.*, 1976*a*). The $Fv\text{-}1^n$ permissive status of CBA is confirmed indirectly (the AKR × CBA is $Fv^{n \times n}$) by the large amount of virus present in the F1 mice. It remains to define the site and activity of what now appears to be a non-oncogenic viral host factor, responsible for the lack of virus in the $Fv\text{-}1^{nn}$ CBA mice, even after embryo transplantation from the AKR strain.

### REFERENCES

Barnes, R. D. & Tuffrey, M. (1974*a*) Absence of Lymphomas in CBA Mice Derived by Embryo Transfer and Born from Lymphoma-prone AKR Mice. *Eur. J. Cancer*, **10**, 575.

Barnes, R. D. & Tuffrey, M. (1974*b*) Lymphoma Susceptibility of the AKR Mouse Strain Acquired before the Stage of Implantation. *Br. J. Cancer*, **29**, 400.

Barnes, R. D., Tuffrey, M., Crewe, P., Dawson, L., Brown, K. & Joyner, J. (1976*a*) High Levels of Oncogenic Virus in Lymphoma Resistant (AKR × CBA)F1. *Cancer Res.* (in press).

Barnes, R. D., Tuffrey, M. & Ford, C. E. (1973) Suppression of Lymphoma Development in Tetraparental AKR Mouse Chimaeras Derived from Ovum Fusion. *Nature, New Biol.*, **244**, 282.

Barnes, R. D., Tuffrey, M., Holliday, J., Hilgers, J. H. M. & Souissi, T. (1976*b*) Murine Leukemia Virus Group Specific Antigen in Tumour-resistant Tetraparental AKR↔CBA/H-T6 Chimaeras. *Br. J. Cancer*, **34**, 28.

Barnes, R. D., Tuffrey, M. & Kingman, J. (1972*a*) The Delay of Leukaemia in Tetraparental Ovum Fusion-derived AKR Chimaeras. *Clin. exp. Immun.*, **12**, 541.

Barnes, R. D., Tuffrey, M., Kingman, J. & Risdon, R. A. (1972*b*) The Disease of the NZB Mouse. Examination of Exchange Ovum Transplantation Derived NZB and CFW Mice. *Clin. exp. Immun.*, **10**, 493.

Ford, C. E., Evans, E. P., Burtenshaw, M. D., Clegg, H., Barnes, R. D. & Tuffrey, M. (1974) Marker Chromosome Analysis of Tetraparental AKR↔CBA-T6 Mouse Chimaeras. *Differentiation*, **2**, 321.

Furth, J., Seibold, H. R. & Rathbone, R. R. (1933) Experimental Studies on Lymphomatosis of Mice. *Am. J. Cancer*, **19**, 521.

Hartley, J. W., Rowe, W. O. & Huebner, R. J. (1970) Host-range Restrictions of Murine Leukaemia Viruses in Mouse Embryo Cell Cultures. *J. Virol.*, **5**, 221.

Lilly, F. (1966) The Inheritance of Susceptibility to the Gross Leukaemia Virus in Mice. *Genetics*, **53**, 529.

McDevitt, H. O. & Benacerraf, B. (1969) Genetic Control of Specific Immune Responses. *Adv. Immun.*, **11**, 31.

Murphy, E. D. (1966) Characteristic Tumours. In *Biology of the Laboratory Mouse*. New York: The Blakiston Div/McGraw-Hill Book Co. p. 521.

Reynolds, E. S. (1973) The Use of Lead Citrate at High pH as an Electron-opaque Stain in Electron Microscopy. *J. Cell Biol.*, **17**, 208.

Sabatini, D. D., Bensch, K. & Barrnett, R. J. (1963) Cytochemistry and Electron Microscopy. The Preservation of Cellular Ultrastructure and Enzymatic Activity by Aldehyde Fixation. *J. Cell Biol.*, **17**, 19.

Tuffrey, M., Barnes, R. D., Evans, E. O. & Ford, C. E. (1973) Dominance of AKR Lymphocytes in Tetraparental AKR↔CBA-T6T6 Chimaeras. *Nature, New Biol.*, **243**, 207.

Watson, M. L. (1958) Staining of Tissue Sections for Electron Microscopy with Heavy Metals. *J. biophys. biochem. Cytol.*, **4**, 475.

Wills, E. J., Tuffrey, M. & Barnes, R. D. (1975) C-type Murine Leukaemia Virus Particles in Tetraparental AKR↔CBA Chimaeras. *Clin. exp. Immun.*, **20**, 563.